# Thermoplastic Elastomer Biocomposites Filled with Cereal Straw Fibers Obtained with Different Processing Methods—Preparation and Properties

**DOI:** 10.3390/polym11040641

**Published:** 2019-04-09

**Authors:** Justyna Miedzianowska, Marcin Masłowski, Krzysztof Strzelec

**Affiliations:** Institute of Polymer & Dye Technology, Lodz University of Technology, Stefanowskiego 12/16, 90-924 Lodz, Poland; justyna.miedzianowska@edu.p.lodz.pl (J.M.); krzysztof.strzelec@p.lodz.pl (K.S.)

**Keywords:** straw, polymer biocomposites, processing

## Abstract

This work is focused on thermoplastic elastomers composites (TPEs) reinforced with straw. Crop waste with different particle size was used as a filler of ethylene-octene rubber (EOR). Application of cheap and renewable natural fiber like straw into a TPE medium is not fully recognized and explored. The effect of fiber orientation induced by two processing techniques on the different mechanical properties of composites was investigated. Microscopic images were used to present the tested straw fractions and observe the arrangement and dispersion of fibers in the polymer matrix. It was found that the usage of an injection molding process allowed for the forming of a more homogenous dispersion of short fiber particles in the elastomer matrix. An oriented straw filler and polymer chains resulted in the improved mechanical strength of the whole system as evidenced by the obtained values of tensile strength almost two times higher for injected composites. In addition, all composites showed very good resistance to thermo-oxidative aging, where the aging factor oscillated within the limits of one, regardless of the processing method and the amount of bioadditive used. On the other hand, vulcanized composites were characterized by greater tear resistance, for which Fmit values increased by up to 600% compared to the reference sample.

## 1. Introduction

Thermoplastic elastomers (TPEs) are gaining more attention in the scientific community since they were first produced nearly 60 years ago. They can be divided into six main types: styrenic thermoplastic elastomers, multiblock copolymers, hard polymer-elastomer combinations, graft copolymers, ionomers, and core–shell morphologies [1]. Thermoplastic elastomers are materials combining the properties of elastomeric and thermoplastic polymers through crystalline hard and amorphous soft phases. Soft segments are responsible for elastic and reversible properties, while hard segments create physical transverse bonds arising as a result of polar interactions, hydrogen bonds, and crystallization [2]. TPEs can be recycled to obtain completely reversible polymers and TPEs’ unique structure provides above-average mechanical properties. They also demonstrate high resistance to oil and heat, as well as good chemical resistance. Processing of TPE materials can be carried out using standard methods such as extrusion, injection molding, or molding and vulcanization. Summarizing, thermoplastic elastomers are multi-functional materials with unique and eco-friendly characteristics, they are recyclable with re-molded and re-shaped possibilities. The global thermoplastic elastomers market was estimated to be 3.82 million tons in 2014. TPE products are widely used in many specific applications including transportation, footwear, industrial goods, wire insulation, medical, adhesives, and coatings [3,4,5].

Notwithstanding the requirements for polymeric material composites, both usability and environmentally friendly properties force scientists to implement new solutions to meet their expectations. One of the methods to improve the characteristics of thermoplastic elastomers is the addition of natural fibers, such as flax, hemp, abacus, sisal, coconut fibers, and kenaf [6]. Different advantages of natural fibers, such as high strength, low weight, corrosion resistance, low maintenance costs low cost, low density, non-abrasive for equipment, non-irritating to the skin, reduced energy consumption, lower health risk, renewable, recyclability and biodegradability, will allow fiber/polymer composites to expand their application in the near future [7,8,9]. Natural fibers are generally unstable above 200 °C, which determines the choice of matrix used in the composite, which is why the most commonly used thermoplastics are polypropylene (PP), polyethylene (PE), and polyvinyl chloride (PVC), and in the case of thermosetting centers, phenolic, epoxy, and polyester resins [10,11,12]. Literature review on the use of natural fibers as additives to composites from elastomeric materials [13,14,15] and thermoplastic elastomers [16,17,18] confirms that the presented topic is not completely exhausted and it is worth looking for new research aspects of these very interesting and future-oriented materials.

An additional, novel aspect of this research is the use of agricultural waste in the form of a cereal straw as a filling for the thermoplastic elastomer matrix. Extensive research interest in this material results from the cellulose, hemicellulose, and lignin in the chemical structure of fibers [19]. So far, straw has been successfully used as a reinforcing material for thermoplastic and resin polymer matrices, mainly due to the fact that it contains about 30–50% cellulose [20,21]. Thermoplastic composites based on straw are generally prepared by melt blending and then by injection, extrusion, or compression [22,23,24,25]. The obtained mechanical properties of the composites proved their usefulness as an alternative to wood or other fiber composites of similar density [26,27]. Similarly, the inclusion of wheat straw can significantly reduce the cost of the product and can be used as a direct alternative to expensive bast fibers. Transformation of wheat straw surplus into innovative, high-performance and cheap market materials certainly has a positive impact from the environmental and industrial points of view.

The purpose of the presented article was to characterize biocomposites filled with agricultural and post-production waste in the form of cereal straw. As a polymer matrix, a thermoplastic elastomer (ethylene-octane copolymer—ENGAGE™) was used. A thorough analysis of materials obtained by two common and widely used industrial processing techniques, injection (without vulcanization) and compression molding (using chemical crosslinking), were carried out. In comparison to a literature review, this presented approach is characterized by multithreading and concerns several aspects of novelty, both in terms of science (new matrix, not fully recognized biofiller) and application (comparison of materials obtained by various preparation methods).

## 2. Materials and Methods

### 2.1. Materials

- Polymer

ENGAGE™ polyolefin elastomer: ethylene-octene rubber (EOR) containing 25 wt.% co-monomer octene was obtained from DOW Chemical Company (Midland, Michigan, USA). The Mooney viscosity was (ML (1 + 4) at 121 °C: 35).

- Crosslinking agent

Rubber mixtures were vulcanized with dicumyl peroxide DCP (purity: 98%) produced by Sigma Aldrich (St. Luis, Missouri, USA).

- Fillers

Cereal (wheat, oat, rye, barley, and triticale) straw was collected from local farms. Dried straw was crushed using a blixer (Blixer 4, Robot Coupe, Vincennes, France) with a grinding time of 20 min at a speed of 3000 rpm. Then sieve analysis was performed by using: vibratory shaker, set of sieves with 2.0; 1.0; 0.5; 0.25 mm nominal mesh size. In following studies, the fractions: 1.0–0.5; 0.5–0.25; 0.25 mm were used. 

The compositions of ethylene-octene copolymer mixtures intended for vulcanization (Table 1) and injection process (Table 1) are presented in Table 1.

### 2.2. Methods

Elastomer mixtures, based on ethylene-octene rubber and straw (Table 1), were prepared using an internal mixer (Brabender Measuring Mixer N50) and next milled with a curing system (DCP) in a laboratory two-roll mill, with roll dimensions of D = 140 mm and L = 300 mm. The rotational speed of the front roll was V_p_ = 16 min^−1^, the friction and the width of the gap between rollers were 1–1.2 and 1.5–3 mm, respectively. The average temperature of the rolls was of about 30 °C.

The kinetics of rubber vulcanization as well as rheometric properties of compounds were studied using a moving die rheometer (Model: MDR) from Alpha Technologies (Hudson, Ohio, USA) (ISO 6502: Rubber-Guide to the use of curemeters) at 160 °C. Determination of minimum torque (M_L_); maximum torque (M_H_); torque increase (dM); scorch time (t_s2_); and the time required for the torque to reach 90% of the maximum achievable torque (t_90_), which is used as an indicator of optimum time cure, were taken from vulcanization curve.

The vulcanization of the rubber mixtures was performed using steel vulcanization molds placed between the shelves of an electrically heated hydraulic press. The samples were cured at 160 °C, and at a 15 MPa pressure for curing time, which was measured by a rheometer.

The crosslinking density of the vulcanizates was determined by equilibrium swelling in toluene, based on the Flory–Rehner equation [28] (Equation (1)):

(1) $$\gamma_{e}=\frac{\ln\left(-1-V_{r}\right)+V_{r}+\mu V_{r}^{2}}{V_{0}\left(V_{r}^{\frac{1}{3}}-\frac{V_{r}}{2}\right)}$$

where *γ_e_*—the crosslinking density (mol/cm^3^), *V*_0_—the molecular volume of solvent (106.7 cm^3^/mol), *µ*—the Huggins parameter of the EOR-solvent interaction calculated from Equation (2) [29]:

(2) $$\mu =\mu_{0}+\beta \cdot V_{r}$$

*µ*_0_—the parameter determining non-crosslinked polymer/solvent relations, *β*—the parameter determining the crosslinked polymer/solvent relations (*µ*_0_ = 0.478, *β* = 0.404),

*Vr*—the volume fraction of the elastomer in the swollen gel (Equation (3)) [28]:

(3) $$V_{r}=\frac{1}{1+Q_{w}\frac{\rho_{k}}{\rho_{r}}}$$

*Q_w_*—the weight of equilibrium swelling, *ρ_k_*—the density of rubber (0.87 g/cm^3^ ), *ρ_r_*—the density of the solvent (0.86 g/cm^3^).

Plate samples injected with different straw content (Table 1) were obtained by means of a PLUS 350, Battenfeld (Vienna, Austria) injection molding machine with injection pressure: 150 MPa, holding pressure: 75 MPa, clamping force: 350 kN, injection time: 3 s, injection rate: 57 cm^3^/s, injection temperature: 160 °C, L/D screw ratio: 14, injection volume: 49 cm^3^, and cooling time: 30 s.

Mechanical properties (tensile strength) of the composites were examined using a universal testing machine Zwick (RoellGroup, Ulm, Germany), at room temperature with a crosshead speed of 500 mm/min for five dumbbell samples for each composite. The measurements of the composites were tested according to the standard procedures in ISO 37. Tear strength tests were carried out using a universal testing machine Zwick (RoellGroup, Ulm, Germany) in accordance with the ISO 34 standard. Dimensions of the samples: 100 mm × 15 mm, “trousers” shape, and test speed: 50 mm/min. 

The thermo-oxidative degradation of the composites was performed at a temperature of 70 °C for 14 days. To estimate the resistance of the samples to aging, their mechanical properties after aging were determined and compared with the values obtained for samples before the aging process. The aging factor (K) was calculated as the numerical change in the mechanical properties of the samples upon aging (Equation (4)) [30]:

K = (TS × EB)_after aging_/(TS × EB)_before aging_ (4)

where: TS is the tensile strength of the sample, and EB is the elongation at break.

The hardness of composites was determined according to the ISO 868 standard using a Shore type A Durometer (Zwick/Roell, Ulm, Germany) and showed average results in ten random points for each sample.

The morphology of straws and composites were examined using an optical stereomicroscope Leica MZ6 (Wetzlar, Germany).

## 3. Results and Discussion

Thermoplastic elastomers could be processed using all methods used for conventional polymeric materials. Their universal character allowed for the use of processing methods, both for thermoplastics, e.g., injection molding, as well as elastomers, e.g., vulcanization.

At the beginning of the study, the processing characteristics of vulcanized TPE biocomposites containing cereal straw of various particle size were made. In the further part of the work, mechanical properties (i.e., tensile strength, tear resistance, hardness) of vulcanized composites with injected non-crosslinked composites were compared. 

**Figure 1:**
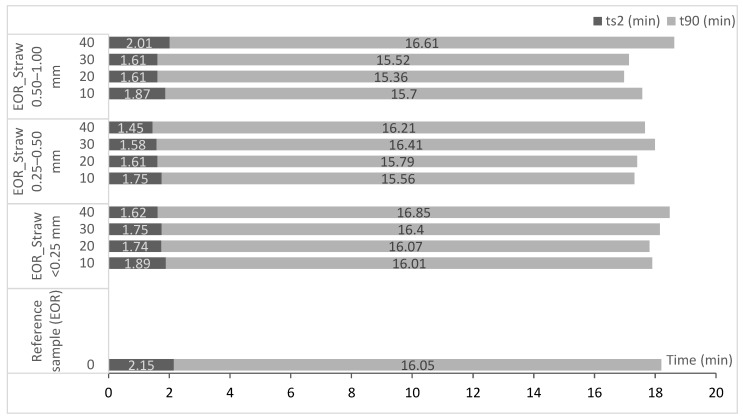
The values of optimum cure time and scorch time of vulcanized composites.

On the basis of vulcametric curves, the rheological parameters, as well as the vulcanization and the scorch time of polymer blends, were determined (Figure 1).

The introduction of straw into the polymer did not significantly affect the values of t_90_ and t_s2_. These parameters showed different results, which oscillated at the level of values obtained for pure ethylene-octene rubber.

The rheometric properties of the rubber compounds are shown in Table 2.

The minimum rheometer torque for all straw-filled rubber compounds increased as compared to the reference sample. The ML value is a measure of the viscosity of the mixture. The analysis of the data showed that mixtures with the smallest particle size filler were characterized as having the lowest viscosity. Regardless of the size of the filler, the minimum torque increased with the increasing content of lignocellulosic material in the composite. The addition of the filler also increased the stiffness of the composites, as evidenced by the increase in the M_H_ value as compared to pure EOR rubber. The use of straw as a filler of elastomeric thermoplastic composites also resulted in a significant increase in torque during rheological tests. The dM value is indirectly related to the crosslinking density of the polymeric material. Smaller-sized straw composites showed higher values of torque increase. Confirmation of the results obtained from rheometric measurements were obtained as studies of equilibrium swelling (Table 3).

For all composites containing straw as a bio-filler, an increase in the spatial concentration of the network was observed. Interactions at the filler-polymer boundary may have contributed to the creation of a more developed structure and affect the spatial structure of the composite. Smaller filler particles, due to larger specific surface area, show increased interfacial adhesion and tendencies to create physical network nodes. A strongly developed filler structure in the polymer matrix should result in a better reinforcing effect.

The main factors affecting mechanical performance of NFCs are:

- fiber selection—including type, harvest time, extraction method, aspect ratio, treatment and fiber content, and matrix selection;
- interfacial strength;
- fiber dispersion;
- fiber orientation;
- composite manufacturing process; and
- porosity [9].

The introduction of fibers of various contents and sizes into the polymer had a significant impact on their dispersion, tendency to agglomerate, and interphase interaction. In contrast, the use of different methods of producing composites resulted in a different arrangement of fibers in the polymer matrix, which also played an important role in the process of strengthening NFC composites.

**Figure 2:**
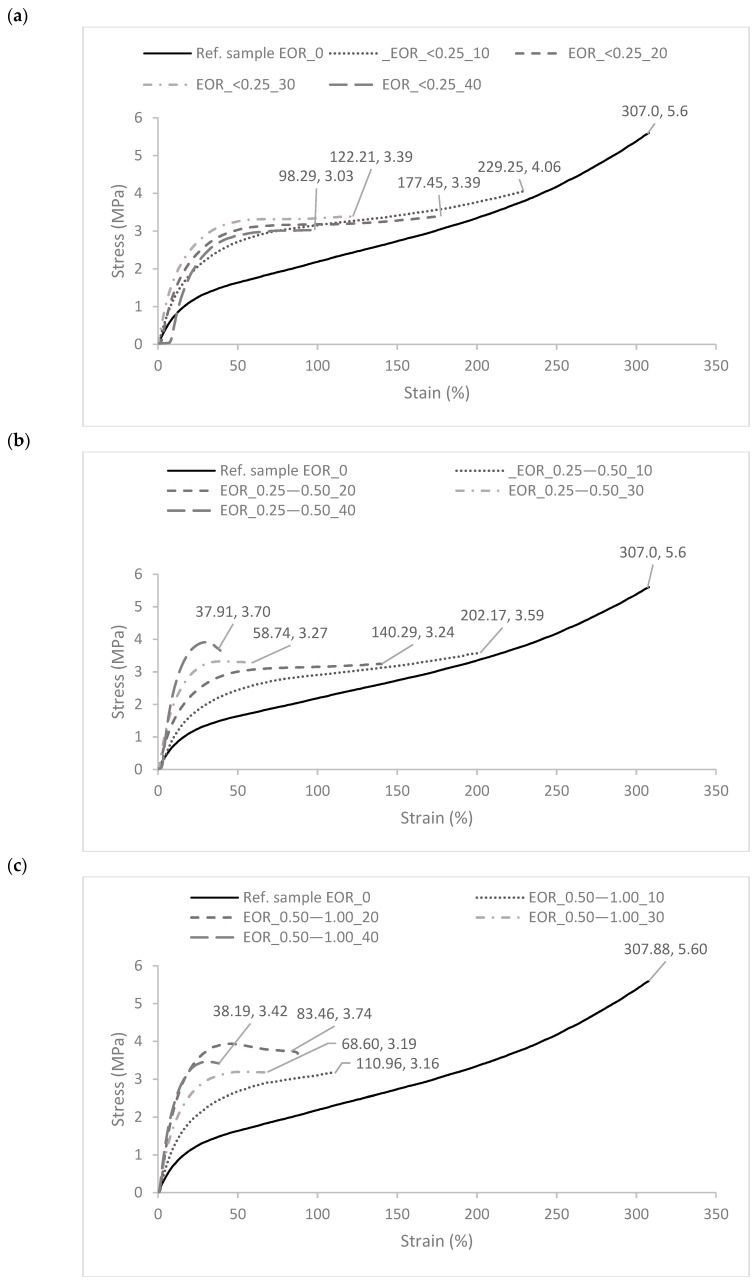
The stress–strain curves of polymer composites containing straw particles of various sizes: (**a**) <0.25, (**b**) 0.25–0.5, and (**c**) 0.5–1.0 mm.

**Figure 3:**
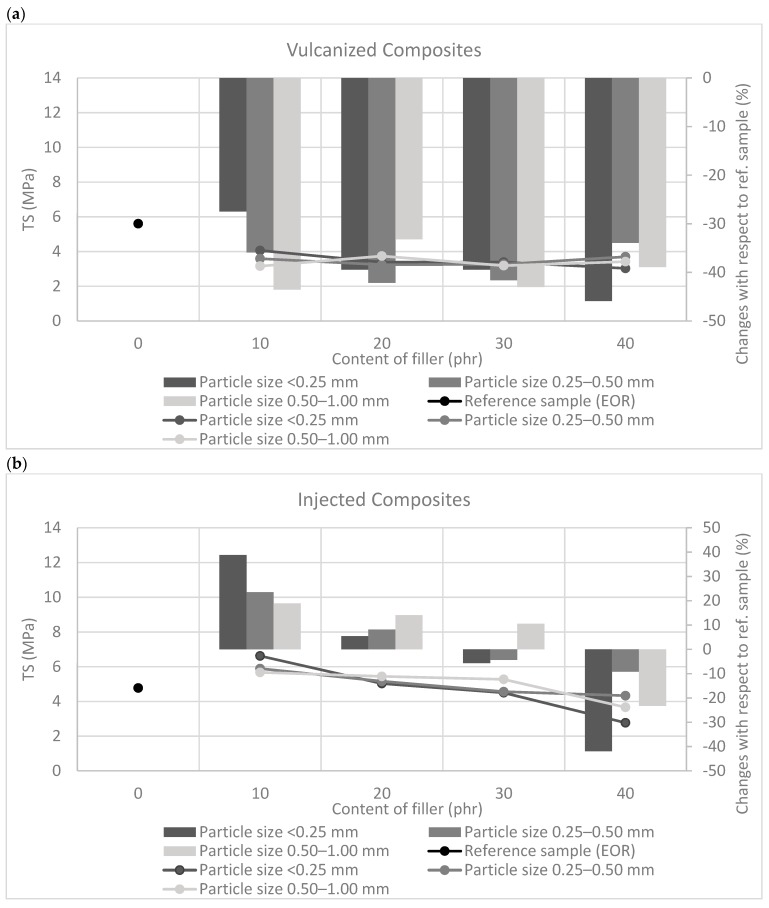
The tensile strength of rubber composites prepared using (**a**) compression molding combined with vulcanization and (**b**) injection process.

The analysis of mechanical properties is presented in Figure 2 and Figure 3. 

The mechanical strength of vulcanized and injected ethylene-octene rubber was 5.6 and 4.8 MPa, respectively. The crosslinking process contributed slightly to the increase in the TS value of the samples, which was probably the result of combining the rubber macromolecules with lateral bonds, which in turn improved the mechanical properties.

In the case of vulcanized composites containing straw (Figure 3a), a decrease in the strength of the samples when breaking was observed. Regardless of the size and straw content, TS values of vulcanizates were approx. 3–4 MPa, which indicates a deterioration of mechanical properties by approx. 30–40% compared to the reference sample. However, for injected composites filled with lignocellulosic material (Figure 3b), in most cases, an improvement in tensile strength was observed. Only composites filled with 40 phr showed a slight deterioration of mechanical properties compared to the unfilled system. The highest increase in the TS value was obtained for the composite containing the straw with the smallest particles. A further increase in the filler content resulted in a reduction in mechanical strength, probably due to the agglomeration and aggregation of bio-filler particles. The improvement of the mechanical properties of the injection composites may have been related to the orientation of the straw particles in the manufacturing process. The straw fibers present in the polymer matrix after passing through the long forming channel are ordered along the direction of flow. As a result of the injection process, composites exhibiting anisotropy of the structure were obtained, which may have influenced the strength properties. At this point, however, it should also be emphasized that from a recent TPE study using injection molding [31], it follows that high shear rate may be responsible for developing fine nanostructured TPE morphology and improved mechanical properties relative to the compression molding process. It is therefore likely that the high shear rate of injection molding is responsible for such an orientation of the straw particles in the manufacturing process. The improvement of mechanical properties of the obtained composites may have a double origin. Therefore, there is a probability of a synergistic effect arising both from the orientation of polymer macromolecules and the natural fibers contained in it during injection as well as the processing parameters occurred while composites are being prepared (high shear rate).

Obtained values of elongation at break varied from 307% for vulcanized to 228% for injected reference samples (Table 4). According to literature data, composites with high elongation (>100%) and low set (<50%) can be included in the group of thermoplastic elastomers [32]. One of the criteria for classifying such materials was thus fulfilled. The addition of lignocellulosic materials in the form of straw, especially in higher content, resulted in a reduction of the elongation at break. This is related to the introduction of a rigid solid phase into the composites, what affected the structure, as well as the mechanical characteristics of the whole system. 

The change in mechanical properties of composites before and after the thermo-oxidative aging simulation was used to calculate the aging factor K (Table 5). If this coefficient is closer to 1, the greater is the material’s resistance to aging processes, resulting in a smaller difference in mechanical properties of composites.

The reference samples, regardless of the method of production, were characterized by an aging factor below 1 (the values were 0.94 and 0.88, respectively), which indicates a slight deterioration of mechanical properties of the samples after thermo-oxidative aging. Vulcanized composites containing straw showed the aging coefficient above unity which indicates the improvement of mechanical strength of the material after the simulation of the aging process. This is probably due to the influence of elevated temperature, which initiated further polymer crosslinking reactions. Physical properties of sulfur-cured rubber vulcanizates depend on their crosslinking densities. The crosslinking densities of rubber vulcanizates cured using a sulfur-accelerator system were changed by thermal ageing. A change of crosslinking densities occurs by formation of new crosslinks and dissociation of existing crosslinks [33]. During the aging process, rubber usually becomes hard and brittle due to the predominant oxidation and crosslinking reactions. The carbon atoms adjacent to the double bonds are easily attacked and form radicals that initiate oxidation and crosslinking [34]. 

Higher values of the K coefficient in comparison to the unfilled system also showed injected composites. Straw as a lignocellulosic material is characterized by a high content of lignin and thus of phenolic acid derivatives in the fiber. These compounds present an antioxidant effect, positively affecting the material’s resistance to degradation process. 

**Figure 4:**
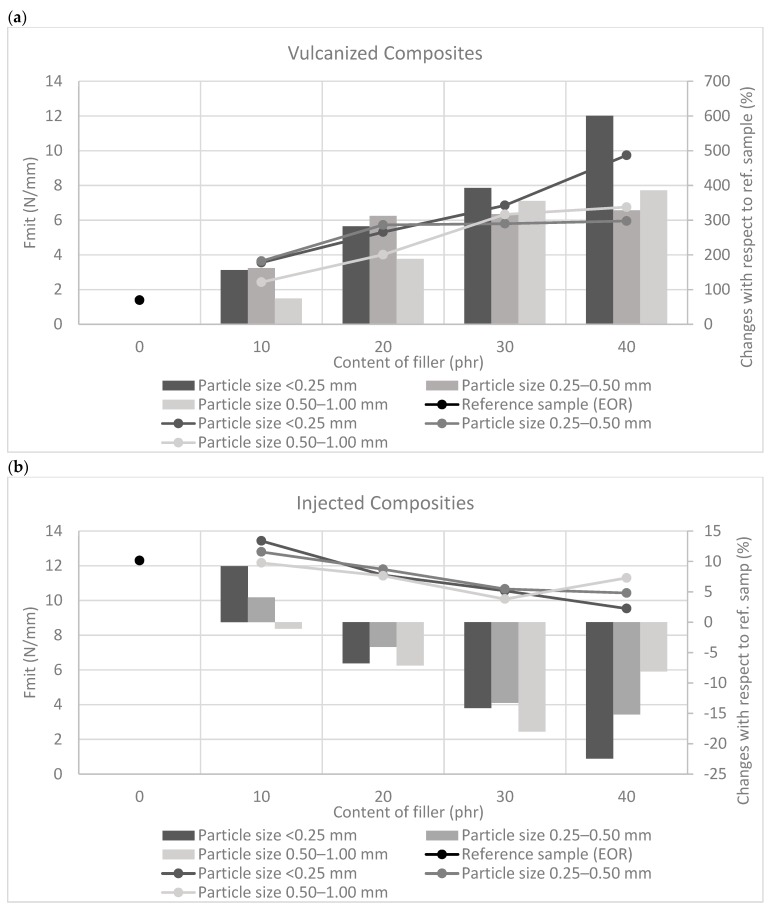
Tear resistance of rubber composites prepared using (**a**) compression molding combined with vulcanization and (**b**) injection process.

The tear strength of vulcanized composites (Figure 4), in which the distribution of filler particles was random and did not have an ordered structure, was greater than in the case of injection-molded samples. The injection process influenced the arrangement of straw particles in the composite, which were characterized by the orientation of the fibers in the direction of the material flow. In the case of injected samples, the tear strength measurement was carried out along the distribution of straw fibers. The morphology of the tested composites may have been the cause of diversified strength properties of materials produced using various methods. When measuring mechanical strength for tearing, filler particles form a barrier limiting material damage. In the case where the particles did not show orientation, they were set at different angles to the tearing force, increasing the stress needed to overcome them. The higher the content of the filler in the test sample, the greater the force required to destroy the material. In contrast, ordered particles, which were arranged in accordance with the direction of the tear, propagated this process, reducing the total material strength to tearing. The use of short grain straw fibers, regardless of the processing method used, improved the tear resistance of the filled systems.

The hardness of both pure rubber and composites containing straw was greater for materials prepared using injection molding (Table 6). The orientation of the filler fibers and the improvement of their dispersion in the polymer matrix increased the stiffness of the material and its hardness. The size of the straw fibers used had a varied effect on the hardness values. The addition of straw particles into EOR decreased the flexibility or elasticity of polymer chains, resulting in more rigid composites. Many works confirmed these conclusions [18,35] and reported that the presence of natural fiber in the thermoplastic elastomer resulted in higher Shore hardness. Among the tested composites, the highest hardness was found in samples containing straw with the smallest particle fragmentation, which may be caused by the highest crosslinking density (vulcanized composites) and a strongly developed filler structure in the polymer matrix. Generally, the hardness of composites increased with higher filler content. The hardness of vulcanized composites was in the range of 67–80 °Sh A, and for injection composites 72–82 °Sh A.

**Figure 5:**
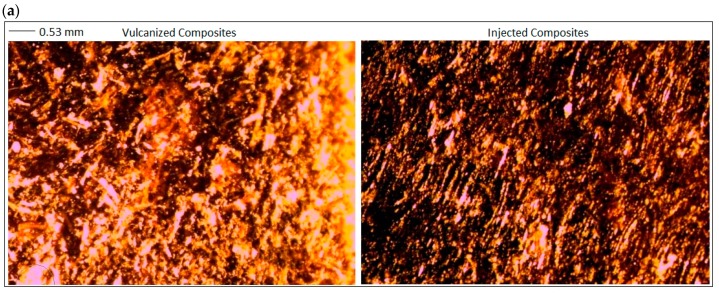

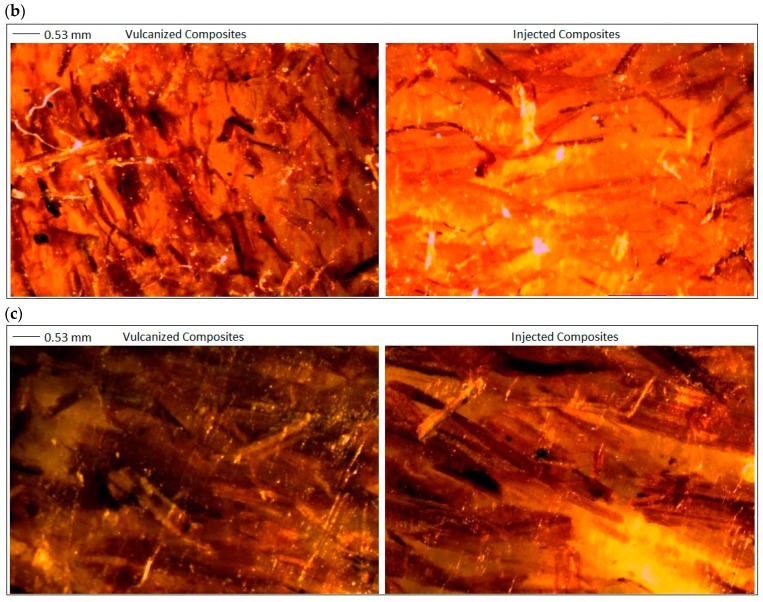
Microscopic photos of composites prepared by compression molding combined with vulcanization (left) and injection process (right) containing straw particles of various sizes: (**a**) <0.25, (**b**) 0.25–0.5, and (**c**) 0.5–1.0 mm.

The process of producing composites containing cereal straw fibers affected the orientation and degree of the dispersion of the filler in the polymer matrix (Figure 5). Microscopic photos of composites prepared using various processing methods, containing filler particles of various sizes, are shown in Figure 5. The use of an injection molding method allowed for creating an ordered secondary structure. In addition, the images shown in Figure 5a for straw-containing composites with a particle size less than 0.25 mm indicate a tendency for agglomeration of the filler in the case of vulcanized samples. Injection-molded composites showed a much more homogeneous dispersion of the filler. During the injection process, the polymeric material flowed through a long channel of the plasticizing system and the injection nozzle [36]. Under the influence of the acting pressure, the fibers contained in the plasticized polymer mass were orientated in the direction of flow. The use of the compression molding technique combined with the vulcanization of the composites led to the production of composites characterized by an isotropic distribution of the filler in the polymer matrix.

## 4. Conclusions

In this work, injection- and compression-molded thermoplastic rubber composites with various straw fiber content and size were prepared. The aim of this research was to recognize, examine, and compare the properties of the materials, in particular, mechanical properties of composites based on the ethylene-octene copolymer, that were processed in two different ways.

1. The differences between rheometric properties and kinetic characteristics of ethylene-octene copolymer mixtures containing straw of different sizes and content/filling were insignificant.
2. Straw-filled rubber mixtures indicated a growth in crosslinking density, and small filler particles created an extended secondary structure in the elastomer, thus contributing to an increase in the concentration of network nodes.
3. The application of an injection molding process for producing composites containing cereal straw fibers positively affected the orientation and degree of the dispersion of the filler in the polymer matrix. Composites characterized by anisotropy of the structure exhibited an improved tensile strength. On the other hand, vulcanized composites with the non-homogeneous distribution of the straw particles in the polymer were characterized by a greater tear resistance.
4. Composites containing biofillers, regardless of the straw type and its size, showed an increased resistance to thermo-oxidative degradation processes.

## Figures and Tables

**Table 1:** The composition of rubber mixtures intended for vulcanization and injection process.

**Polymer: EOR (phr)**	**Filler: Straw (phr)**	**Crosslinking Agent: DCP (phr)**
100	-	2
Straw with particle size 1.0–0.5 mm		
100	10	2
100	20	2
100	30	2
100	40	2
Straw with particle size 0.5–0.25 mm		
100	10	2
100	20	2
100	30	2
100	40	2
Straw with particle size < 0.25 mm		
100	10	2
100	20	2
100	30	2
100	40	2
**Polymer: EOR (phr)**	**Filler: Straw (phr)**	
100		
Straw with particle size 1.0–0.5 mm		
100	10	
100	20	
100	30	
100	40	
Straw with particle size 0.5–0.25 mm		
100	10	
100	20	
100	30	
100	40	
Straw with particle size < 0.25 mm		
100	10	
100	20	
100	30	
100	40	

phr: parts per hundred rubber.

**Table 2:** Rheometric parameters of the EOR compounds.

Sample Name	Content of Filler (phr)	M_L_ (dNm)	M_H_ (dNm)	dM (dNm)
Reference sample (EOR)	0	0.68	8.88	8.2
EOR_Straw < 0.25 mm	10	0.98	11.04	10.06
	20	1.21	12.84	11.63
	30	1.32	14.01	12.69
	40	1.37	15.96	14.59
EOR_Straw 0.25–0.50 mm	10	1.09	11.67	10.58
	20	1.39	14.06	12.67
	30	1.43	14.83	13.4
	40	1.72	16.63	14.91
EOR_Straw 0.50–1.00 mm	10	1.09	10.96	9.87
	20	1.34	13.27	11.93
	30	1.33	14.42	13.09
	40	1.43	15.16	13.73

**Table 3:** The crosslinking density of the EOR-filled vulcanizates.

ν_e_ (×10^−5^) (mol/cm^3^)					
Content of filler (phr)	0	10	20	30	40
Reference sample (EOR)	2.53				
EOR_Straw < 0.25 mm	-	3.09	3.57	4.32	5.42
EOR_Straw 0.25–0.50 mm	-	3.15	3.62	3.83	4.24
EOR_Straw 0.50–1.00 mm	-	2.75	3.28	3.79	3.85

**Table 4:** The elongation at break of EOR biocomposites.

Sample Name	Content of Filler	Vulcanized Composites	Injected Composites
		Eb	Eb
	(phr)	(%)	(%)
**Reference sample (EOR)**	0	307	228
**EOR_Straw < 0.25 mm**	10	229	269
	20	177	211
	30	122	207
	40	98	173
**EOR_Straw 0.25–0.50 mm**	10	202	249
	20	140	215
	30	58	189
	40	37	136
**EOR_Straw 0.50–1.00 mm**	10	110	220
	20	83	219
	30	68	234
	40	37	159

**Table 5:** The aging factor (K) of straw-filled composites.

K (-)					
Vulcanized Composites					
Content of filler (phr)	0	10	20	30	40
Reference sample (EOR)	0.94				
Particle size < 0.25 mm		1.13	1.23	1.20	1.21
Particle size 0.25–0.50 mm		1.24	1.11	1.16	1.22
Particle size 0.50–1.00 mm		1.29	1.25	1.13	1.17
Injected Composites					
Content of filler (phr)	0	10	20	30	40
Reference sample (EOR)	0.88				
Particle size < 0.25 mm		0.96	0.99	1.00	1.04
Particle size 0.25–0.50 mm		1.11	1.02	0.99	1.06
Particle size 0.50–1.00 mm		1.05	1.05	0.99	1.01

**Table 6:** The hardness value of EOR composites containing straw fibers.

Hardness (°Sh A)					
Vulcanized Composites					
Content of filler (phr)	0	10	20	30	40
Reference sample (EOR)	64.32				
Particle size < 0.25 mm		70.02	74.62	78.83	80.22
Particle size 0.25–0.50 mm		67.13	67.90	68.00	79.08
Particle size 0.50–1.00 mm		67.12	73.30	78.57	80.67
Injected Composites					
Content of filler (phr)	0	10	20	30	40
Reference sample (EOR)	70.30				
Particle size < 0.25 mm		76.02	79.21	80.41	82.14
Particle size 0.25–0.50 mm		72.32	75.55	79.03	80.85
Particle size 0.50–1.00 mm		73.52	75.05	80.25	82.11
